# Complications from carcinoid syndrome: review of the current evidence

**DOI:** 10.3332/ecancer.2016.662

**Published:** 2016-08-08

**Authors:** José Mauricio Mota, Luana Guimarães Sousa, Rachel P Riechelmann

**Affiliations:** Instituto do Câncer do Estado de São Paulo, University of São Paulo, 01246-000 Brazil

**Keywords:** carcinoid syndrome, complications, carcinoid heart disease, fibrosis

## Abstract

Patients with well-differentiated neuroendocrine tumours may develop carcinoid syndrome (CS), which is characterised by flushing, abdominal cramps, diarrhoea, and bronchospasms. In this scenario, long-term secretion of vasoactive substances—serotonin, tachynins, and others, may induce fibrogenic responses in local or distant tissues, leading to complications such as carcinoid heart disease (CHD), mesenteric and/or retroperitoneal fibrosis. Rare cases of lung/pleural fibrosis and scleroderma have also been described. Despite it not being well described yet, current evidence suggests the pathogenesis of such fibrogenic complications relies on signalling through 5-HT_2B_ and TGF-β1. Medical management is still very limited and lacks prospective and randomised studies for definitive recommendations. Surgical procedures remain the best definitive treatment option for CHD and abdominal fibrosis. Recently, cognitive impairment has also been described as a potential consequence of CS. This review critically discusses the literature concerning the epidemiology, pathogenesis, clinical features, diagnosis, and treatment options for CS-related long-term complications.

## Introduction

Between 2000 and 2004, well-differentiated neuroendocrine tumours (NET) occurred in 5 per 100,000 people per year in the US according to the Surveillance, Epidemiology, and End Results (SEER) 17 [1]. For unknown reasons–some argue because of improvement of diagnostic imaging methods—the incidence of this type of neoplasm is increasing [1–2]. One of the most common NET is the well-differentiated midgut NET presenting with carcinoid syndrome (CS). This paraneoplastic syndrome usually affects patients with gastrointestinal NET arising from the midgut (i.e. small intestine, appendix, and proximal colon), and less commonly from the lung and the pancreas [3]. Carcinoid cells synthesise, store, and release a myriad of bioactive amines, polypeptides, and lipids, such as 5-hydroxytryptamine, bradykinins, tachykinins, prostaglandins, and histamine [4]. Although the precise pathogenesis remains elusive, these substances are implicated in the development of CS. Tumour metastatic spread to the liver circumvents the hepatic inactivation of these substances and leads to CS development [5–6]. CS seldom occur in the absence of liver metastases, unless tumour products directly drain to the systemic circulation such as in the case of lung NET-associated CS or patent foramen ovale [7].

CS is clinically characterised by cutaneous flushing attacks, hypotension, bronchospasm, abdominal cramps, and diarrhoea [8]. Because patients with well-differentiated midgut NET and CS tend to live for several years, acute and chronic complications from CS if they arise may possibly impact on overall survival(OS) and quality of life (QoL). Furthermore, many patients take several years to be correctly diagnosed with NET. Of note, fibrosis is considered a hallmark of CS. The most known complication of CS is carcinoid heart disease (CHD), characterised by a fibrotic degeneration of cardiac valves and extracardiac fibrosis. The aim of this review is to critically describe the current state of the art in respect to epidemiology, pathogenesis, clinical features, diagnosis, and treatment options for complications associated with CS resulting from neuroendocrine tumours.

## Methods

A search in the Pubmed/Medline database was conducted in order to track relevant papers about complications associated with CS. Keywords ‘malignant carcinoid syndrome’, ‘carcinoid heart disease’, ‘fibrosis’, ‘carcinoid tumours’, ‘neuroendocrine tumours’, ‘complications’, and ‘pathogenesis’ were combined for this purpose. English language was mandatory for paper selection. Eligible studies were case reports, series, retrospective, and prospective studies. Articles were screened and critically analysed. Preclinical studies and historical papers were assessed on an individual basis.

## Carcinoid heart disease

### Epidemiology

The term CHD (Hedinger’s syndrome) refers to the combination of right-sided valve dysfunction associated with a morphological alteration, such as valve leaflet thickening, retraction, and/or insufficient cooptation [9]. CHD occurs as a result of chronic exposure of the heart endothelium to several vasoactive substances, usually after a mean period of 1.5 years from diagnosis of CS [10]. In a large case series of 132 patients with CS, echocardiography assessment detected signs of CHD in 74 patients (56%). The main site of primary tumour was small bowel in 72% of patients, whereas 18% remained as occult primary neoplasia [11]. However, this study was conducted before more new accurate imaging techniques were available, such as the PET-CT gallium 68.

Subsequent case series reported different incidence rates, ranging from 35– 59%, although the criteria used to diagnose CHD was largely variable across the studies [3, 12–13]. A more recent prospective study followed 252 patients with CS and detected a lower incidence of CHD (19.4%) after a median followup of 29 months [14]. Although purely speculative, one might claim that this lower incidence may be resultant of lead-time bias, as patients may have had shorter disease time compared to other studies, or a short follow-up period to observe events. Another possible explanation might be a long-lasting exposure to effective antitumour treatments such as somatostatin analogues.

### Pathogenesis

CHD results from chronic fibrotic degeneration of the cardiac valve leaflets. Despite the fact that the precise pathways involved in this complication remain largely unknown, it was proposed that the vasoactive substances produced and secreted by the tumours induce myofibroblast proliferation and local deposition of extracellular matrix, ultimately leading to the emergence of endocardial plaques composed of matrix-rich fibrous tissues [15–16].

Efforts have been conducted in order to clarify the pathogenesis of CHD. Musunuru and colleagues created a xenograft model of CHD in which nude mice were inoculated with pancreatic carcinoid BON cells and developed fibrotic degeneration of right-sided valve leaflets [17]. An emerging body of evidence has pointed towards serotonin as the possible main causative agent of CHD. First, the levels of serotonin and 24-hour urinary 5-hydroxyindolacetic acid (5-HIAA) were significantly higher in patients who developed CHD when compared with those without CHD [18–19]. Second, many serotonergic drugs such as ergot alkaloids, cabergoline, Ecstasy, and fenfluramine are related to right-sided valvular fibrosis [20]. Finally, preclinical studies have shown that exogenous serotonin administration induces endothelial cell proliferation *in vitro* [21] and subendothelial fibrotic plaque deposition *in vivo* [22]. However, as we will be discussing further, controlling the serotonin production with somatostatin analogues does not seem to impede the development or progression of CHD.

Serotonin-induced cardiopathy possibly occurs through 5-HT_2B_. Activation of this G-protein coupled receptor mediates fibroblasts and smooth muscle cells mitogenic signals, as well as secretion of cytokines and extracellular matrix components [23–24]. In this line, Nebigil and colleagues generated transgenic mice over-expressing 5-HT_2B_ receptors specifically in the heart, resulting in cardiac hypertrophy and extracellular matrix deposition [25]. The same group also demonstrated that knocking-down 5-HT_2B_ receptors led to heart malformations during embryogenesis and ventricular dilation during adulthood [26]. Additionally, signalling through 5-HT_2B_ induces an increased expression of TGF-β1 [27], a key mediator of myofibroblast activation and fibrogenic responses which has also been previously related to CHD [28]. Despite serotonin being important, CHD is believed to be a multifactorial phenomenon, since several other mediators have been associated with CHD. For example, increased levels of activin A, a protein from the TGF-β superfamily, were detected in the serum and endocardial plaques of patients with CHD, independently of disease stage or severity [29]. Other substances such as tachykinins [30] and connective tissue growth factor [31] were related to fibroblasts proliferation, functioning and CHD development.

Regardless of the still unknown precise underlying mechanism, fibrotic plaque deposition usually affects the downstream side of the valve leaflets (i.e. ventricular aspect of tricuspid valve and pulmonary side of pulmonary valve) and subvalvular apparatus [32]. The accumulation of fibrosis leads to a progressive movement restriction, retraction and fixation, which are clinically transduced to right-sided valve stenosis, regurgitation, or a variable combination of both. Right heart failure may occur as a long-term consequence [9]. Interestingly, the left side of the heart is relatively protected from CHD because the vasoactive peptides are inactivated after passing through the lungs before they reach the left atrium. In patients with bronchial carcinoids, a patent foramen ovale or very poor-controlled CS, serotonin, and other CHD-causative substances can bypass lung inactivation, resulting in left-sided CHD development [33].

### Clinical features

Because the pulmonary circulation is a low-pressure system, many patients may tolerate gradual and progressive increases of tricuspid or pulmonary valve stenosis or regurgitation. This possibly explains why a large proportion of patients with CHD remain asymptomatic during the course of the disease. For this reason, relying solely in the clinical assessment is not sufficient to diagnose CHD. In a previous cross-sectional study, 57% of patients with severe CHD, echocardiograph alterations were either asymptomatic or presented with mild symptoms [34].

Studies have shown that CHD is often present in patients whose CS lasted for a minimum period of 1.5–2 years [3]. Fatigability and progressive exertional dyspnoea are usually the first symptoms. Oedema, weight gain, ascites, and upper right abdominal pain because of hepatomegaly point to right heart failure. An important physical finding is external jugular venous distension which can occur in tricuspid regurgitation and right heart failure. Additionally, examination of jugular venous pulse can unravel a large ‘v’ wave in tricuspid regurgitation or a large ‘a’ wave in tricuspid stenosis or right heart failure [35]. Heart auscultation is crucial to detect murmurs of tricuspid/pulmonary regurgitation or stenosis in asymptomatic patients. In a large case series, heart murmurs were detected in 92% of patients with CHD versus 43% of patients without CHD [11].

### Electrocardiogram and chest x-ray

Electrocardiogram and chest-x ray are inaccurate for CHD assessment. Pellikka and colleagues retrospectively evaluated the electrocardiogram findings of 68 patients with CHD and detected no alterations in 31% of cases. ST-T wave abnormalities were found in 24%, whereas sinus tachycardia and low voltage were detected in 13% and 10% respectively. Chest-x ray was normal in 46% and cardiac enlargement was found in only 18% of CHD patients [11].

### Echocardiography and imaging

Echocardiography has been recognised as the principal method to detect CHD and to assess disease severity [9, 36]. Thickening of valve leaflets, chordae, and papillary muscles are apparently the initial findings. As disease progresses, valve leaflets fixation and retraction occur. Tricuspid abnormalities are the most common valve alteration and can occur in up to 90% of subjects with CHD [37]. In 42% of CHD cases, all tricuspid leaflets were thickened and perceived to be in a semi-open fixed position, resulting in tricuspid stenosis and/or regurgitation [37]. Severe tricuspid regurgitation led to volume overload and produced right ventricle (90%) and atrial (100%) dilatation [37]. Abnormalities in pulmonary valve are less often detected (49–69%) in these patients [11, 37].

Rather than solely valvular, the deposition of carcinoid plaques can also occur diffusely, as the transesophageal echocardiography detected endocardial thickening in 90% of CHD subjects [37]. Left-sided valvular disease is less common; in a retrospective series, most cases of left-sided disease were found in patients with patent foramen ovale (87%) and less likely with bronchial carcinoids (13%) [37]. The development of foramen ovale patency in these patients is not negligible and marks CHD progression. A retrospective study showed that after a median follow-up of 24 months, the incidence of patent foramen ovale doubled from 20–41%, with an odds ratio for the association of CHD progression and a foramen ovale of 44.2 [38].

Cardiac magnetic resonance imaging (MRI) is another valuable strategy to assess CHD diagnosis and severity. It can add further information, especially in obese patients with limited acoustic window when echocardiography is very limited. MRI can reveal any hypointense thickening of valve leaflets and subvalvular apparatus with a late enhancement occurring after gadolinium administration. Besides, MRI is an accurate method to measure tricuspid regurgitant volume and right ventricular ejection fraction [39–40].

The European Neuroendocrine Tumour Society (ENETS) guideline recommends performing annual or more often if medically required, transthoracic echocardiography in patients with known or suspected CHD with a ‘bubble study’ to exclude a patent foramen ovale. Because echocardiography is an operator-dependent imaging modality, a personal experience of at least 200 examinations per year is recommended, although this may not be realistic worldwide. For those patients who have inadequate acoustic windows, MRI should be considered [41, 42]. In the Brazilian NET guideline (Riechelmann R *et al*, unpublished data), we also recommend that asymptomatic patients with elevated 5-HIAA, regardless of the presence of CS, perform an echocardiograph test to screen for CHD. If no signs of CHD are found, annual echocardiography is recommended.

### Biochemical evaluation

Currently, there is no specific biomarker that predicts which patients will develop CHD. Studies have shown that patients with CHD have elevated serum and platelet serotonin levels as well as abnormal 24 hour urinary 5-HIAA [19, 43–44]. A linear association between 5-HIAA urinary levels and CHD development and progression has been suggested [3]. In a prospective study of 252 patents with CS who had echocardiograph tests every six months, 24 hour urinary 5-HIAA≥300 μmoL/24 hour and at least three flushing episodes per day were associated with a 2.74 higher risk of onset or progression of CHD [14]. Although unproven, this finding indirectly suggests that controlling the 5-HIAA urinary levels with medical therapy may be beneficial in patients with CHD. Other substances possibly linked to CHD pathogenesis, as activin-A [29] and connective tissue growth factor [31], have been shown to be independent predictors of CHD development. However, laboratory assays to measure them are not usually available in the clinical setting and lack proper validation.

The neurohormone N-terminal pro-brain natriuretic peptide (NT-pro-BNP) reflects ventricular dilation and is widely used to diagnose heart failure and predict its severity. NT-pro-BNP is also an accurate biomarker for CHD development and severity in patients with carcinoid tumours [45–47]. Dobson and colleagues have shown that an increase of 100 ng l^-1^ in serum NT-pro-BNP increased risk of death by 11% [48]. The ENETS consensus recommends periodically monitoring NT-pro-BNP in all patients with CS to promote early detection of CHD [41], despite the fact that NT-pro-BNP is probably not accurate in early cases of CHD, because of its apparent dependency on ventricular dilation.

### Prognosis

A retrospective analysis from 1995–2000 reported a median survival of 4.4 years (95% CI 2.4–7.1 years) in patients diagnosed with CHD. Apparently, survival increased along the years, because mortality was higher in patients with CHD diagnosed from 1981–1989 [49]. Previous studies indicated that patients with CHD had reduced survival when compared with those without CHD [50]. Older patients and those with severe tricuspid regurgitation are at increased risk of death [51]. Tissue Doppler imaging may be an important method to assess mortality risk. Mansencal and colleagues demonstrated that a ratio of early transmitral flow velocity to early diastolic mitral annulus velocity (E/e’ ratio)≥8 was the only independent marker of death detected by the multivariate analysis (odds ratio = 6.2). After a mean follow-up of 34 months, the all-cause mortality rate was significantly higher among patients with the E/e’ ratio was ≥8 (94%) versus those with E/e’ ratio <8 (13%) [52].

### Medical treatment

Once established, CHD-related valvulopathy and cardiac fibrosis is usually not reversible. Patient care should be transitioned to a specialist centre with a multidisciplinary team. Because of its rarity, there are no randomised trials specifically assessing this issue yet. Given the evidence suggesting that serotonin is behind the pathogenesis of CHD and that the higher the 5-HIAA 24 hour urinary levels the higher the risk of having CHD, it is logical to think that maximum control of serotonin levels would prevent or at least delay the onset and progression of CHD. However, the literature is controversial on this topic. An uncontrolled prospective study did not show that the reduction in 5-HIAA achieved by treatment with somatostatin analogs led to regression of established CHD lesions [12]. Retrospective analyses also did not show any difference in CHD progression between patients who were using of somatostatin analogues and those who were not [34, 48]. PROMID, a large prospective randomised placebo-controlled double-blinded trial, which included patients with metastatic midgut NET showed reduced time to tumour progression in the group treated with octreotide LAR *versus* placebo. However, although nearly 40% of patients had CS, development or progression of CHD was not evaluated in this study [53–54]. The RADIANT-2 study was a phase III placebo-controlled trial of octreotide LAR alone or combined with everolimus in patients with functioning well-differentiated NET and CS [55]. Similarly to PROMID, this trial did not provide information of whether patients with CHD were enrolled and if so, what were their outcomes.

Small retrospective series suggest that mechanical tumour debulking may benefit patients with CHD. In a retrospective analysis of 77 patients with CHD and serial echocardiograms, where ten underwent hepatic resection of liver metastases, resection was independently associated with a lower risk of CHD progression (odds ratio = 0.29, 95% CI = 0.06–0.75) [56]. The role of other anti-tumour medical interventions, such as hepatic embolisation and peptide receptor radionuclide therapy in the control of CHD remains to be determined. A new agent, telotristat etiprate, an oral inhibitor of the tryptophan hydroxylase with resultant inhibition of serotonin synthesis was tested in patients with refractory CS. TELESTAR was a double-blinded trial that randomised 135 well-differentiated NET patients to receive either placebo or two different doses of telotristat etiprate (250 mg or 500 mg t.i.d.). Patients had to have at least four bowel movements per day, despite optimum therapy with somatostatin analogues. The primary objective was met, as the study demonstrated a reduction in bowel movements frequency in the groups who received telotristat. Additionally, reduction in 5-HIAA levels were detected in the telostristat arms [57]. Interestingly, two patients enrolled in this study (one of them with imminent need of valve surgery) had their CHD halted with no further fibrosis observed on serial echocardiographic tests [58]. Trials of telotristat etiprate in CHD are highly awaited by the oncology community.

The medical treatment of established CHD relies on palliative measures such as loop diuretics, salt and fluid restriction to reduce oedema. Caution should be exercised in patients with severe right heart failure, because excessive fluid depletion can reduce cardiac output and produce clinical worsening [9]. Recently, novel strategies have been tested and might implicate a better treatment of CHD. A single-arm prospective uncontrolled study of bosentan, a dual endothelin receptor antagonist, in 14 patients with CHD observed that NT-pro-BNP levels, symptoms (NYHA classification) of right ventricular systolic pressure and six-minute walk distance improved after six months of treatment [59].

Because of the aforementioned speculated relevance of 5-HT_2B_ receptors in triggering cardiac fibrotic reactions, studies evaluating the role of inhibiting this pathway to prevent or treat CHD are greatly desired and expected. 5-HT_2B_ inhibitors have a potential value in treating fibrosis resultant from different scenarios. For instance, specific antagonists of serotonin 5-HT_2B_ receptors reduced fibrosis and protected against right ventricle failure in a mice model of pulmonary hypertension [60] and reduced bleomycin-induced lung fibrosis in mice [61].

### Surgical management

The only definitive treatment for advanced CHD is valve replacement surgery or valvuloplasty. Despite the absence of prospective randomised studies, the literature recognises that cardiac surgery remarkably improves symptoms and reduces mortality in patients with severe CHD [9, 62–63]. In the Mayo Clinic retrospective analysis of 200 patients with CHD, when cardiac surgery was included in the multivariate analysis as a time-dependent covariate, it was associated with significant reduced ten-year mortality as opposed of other treatment modalities [49]. However, perioperative mortality is still a relevant issue. Early postoperative 30-day mortality was previously related to as up to 63% [64] in the first series. Better patient selection, advances in surgical techniques, perioperative supportive intensive management, and global multidisciplinary care may be responsible for the noted improvement in perioperative mortality observed along the last decades [49]. In a recent study, mortality rate after cardiac surgery for CHD between 1985 and 2012 was 10%. Considering the period after 2000, perioperative mortality rate was 6%. Older age, tobacco use, and treatment with cytotoxic chemotherapy were associated with greater mortality. Importantly, patients surviving the operation presented with satisfactory rates of symptom control and survival up to 19.5 years [65]. In concordance with these findings, another study reported an inhospital mortality of 7% [66].

Concerns about surgical and anesthetic risks are not negligible. Patients with CHD, especially those with uncontrolled CS, are at increased intraoperative risk. Firstly, patients may present low-left ventricle output which produces a risk of hypotension. Secondly, catecholamines and histamine releasing drugs used in anesthetic and perioperative management can precipitate carcinoid crisis, producing bronchospasm, arrhythmias, and vasoplegia-induced haemodynamic instability [67]. Experts have previously recommended avoidance of long-acting opioids, histamine-releasing neuromuscular relaxants (e.g. atracurium, succinylcholine), inotropes, or vasopressors in anesthetic management of these patients [68]. Despite the low evidence level (expert opinion), anesthetic induction can be done with etomidate and maintained with isoflurane. Muscle relaxation can be performed with rocuronium or vecuronium, and the analgesics used can be fentanyl or sufentanil [69].

Proper indications and timing for valve replacement are still a matter of constant debate. Symptomatic and asymptomatic patients with progressive right-sided heart enlargement or dysfunction may benefit from surgery [62]. For obvious reasons, definitive treatment should be avoided in patients with poorly controlled or end-stage metastatic disease. Attention to an increased risk of bleeding should be paid as it can possibly happen with increased venous pressures because of right heart failure. For that reason, experts have recommended cardiac surgery to be performed before partial hepatectomy or liver transplantation when indicated [9, 70].

There are no comparative studies evaluating the best valve prosthesis type to implant. Rather than promote a general recommendation, we believe that this decision should be taken in an individual in a more tailored basis. The decision should always be made after discussing with specialised multidisciplinary team of oncologists, cardiologists, and cardiac surgeons, taking into consideration many factors such as age, comorbidities, risk of bleeding, life expectancy, availability of further treatment options, patient preferences, and experience of the cardiovascular surgery team. Mechanical prosthesis has the theoretical benefit of being less prone to premature degeneration [71–72]. On the other hand, biological valves do not require permanent anticoagulation and are much more less associated with prosthetic valve thrombosis [73]. A recent retrospective analysis compared survival after tricuspid valve replacement with a nonsignificant reduced mortality in favour of the group who received bioprosthetic valves [65]. Importantly, based on its retrospective views, we see this study is prone to selection biases and confounding factors and does not allow any definitive conclusion in the regard of which valve type is better for CHD patients. Another series showed that combined tricuspid and pulmonary valve replacement appeared to have a beneficial role in suitable patients [74]. Several case series suggest that frailer patients or those with high surgical risk with pulmonary stenosis may temporarily benefit from balloon pulmonary percutaneous valvuloplasty [75–76]. Additionally, previous reports indicated that patients with a patent foramen ovale have symptoms and functional state improved after a percutaneous closure procedure [77].

## Extracardiac fibrosis

Midgut NET-associated CS can lead to local desmoplastic responses around the tumours in the mesentery and peritoneum, with consequent small bowel obstruction, kinking, ischemia/angina, and volvulus. Intestinal obstruction because of the primary tumour is a common first presentation. In a series of 314 patients with midgut carcinoid tumours, 46% were operated on an emergency basis because of intestinal obstruction caused by the primary tumour. Local fibrosis may contribute to this first presentation [78]. Because of the high risk of obstruction associated with local fibrotic reaction, some experts recommend the removal of the primary tumour, independently of local symptoms [41, 79]. Although there is no prospective evidence supporting the resection of primary midgut carcinoid tumours in patients with unresectable metastasis, we advocate in favour of this strategy for patients with good performance status, few comorbidities, and controlled systemic disease in order to avoid the risk of obstruction.

**Figure 1.**
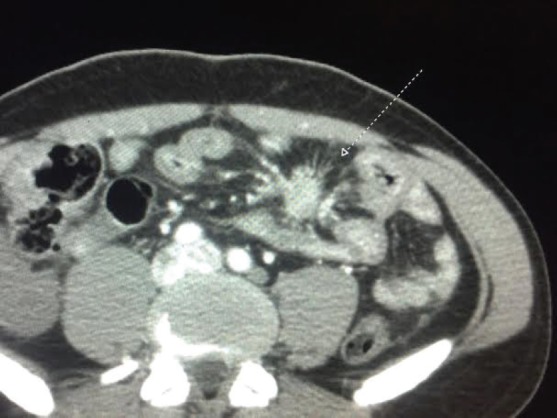
Fibrosis with mesenteric retraction in a patient with CS.

Peritoneal dissemination can also produce marked fibrotic local reactions even in the absence of CS [80–81]. Massive fibrosis can lead to occlusion of mesenteric vessels and small intestinal infarction [82]. In a small series of 31 patients, half presented intra-abdominal fibrosis and a toal of 16 presented with small-bowel thickening, 13 with soft-tissue stranding, 2 had a ‘misty’ mesentery, and 2 had retroperitoneal fibrosis [83]. Figure 1 depicts an example of mesenteric fibrosis in a patient with CS. Additionally, mesenteric masses can appear as sclerosing mesenteritis with the pathognomonic ‘fat ring sign’ on CT scans [80].

Despite its rarity, there are several reports linking carcinoid tumours with retroperitoneal fibrosis [84–85] that can produce stenosis of the ureters and hydronephrosis [84]. One of them reported a reduction of retroperitoneal fibrosis after long-term treatment with octreotide and tamoxifen [86]. Although the precise mechanisms of extracardiac fibrosis have not yet been fully unraveled, serotonin, and TGF-β are believed to be possible mediators. Interestingly, methysergide, a nonselective serotonin antagonist, has been associated with cases of retroperitoneal, endocardial, and valvular fibroses [87].

The association between CS and skin fibrosis was first described in 1958 [88]. Cutaneous scleroderma is a rare and usually late feature of CS characterised by skin thickening and loss of elasticity because of dermal fibrosis [89]. Bell and colleagues reported a series of 25 patients with carcinoid tumours, two of them with scleroderma [90]. Serotonin and other vasoactive substances are believed to be the causative agents of scleroderma as a result of inflammatory response because of recurrent vasospasm and flushing attacks [80–91]. Data is very limited regarding treatment. Previous isolated reports described significant improvement of cutaneous signs after treatment with octreotide [92], cyproheptadine, or prednisolone [89]. A small series of six male patients with CS described that two of them developed plastic induration of the penis (Peyronie’s disease) [93]. Moss and colleagues analysed CT scans of 50 patients with midgut NET and detected pleural thickening in 14 of them. Nine cases were considered idiopathic and could be probably because of CS [94]. Cases suggesting associations between CS and diffuse interstitial pulmonary fibrosis [95] and obliterative bronchiolitis with airflow obstruction in pulmonary tests have been reported [96].

Besides the mesenteric fibrosis associated with the primary tumour, the other types of extracardiac fibroses are fortunately rare in most specialised centres. Likely because there is more awareness about NET, patients are diagnosed earlier; also, because of level 1 evidence that somatostatin analogues prolong time to progression, these agents have been used more often, and thus patients are probably treated more timely. However, likewise for CHD, it is unknown whether the control of CS with medical therapies prevents or delays extracardiac fibrosis.

## Carcinoid crisis

Carcinoid crisis is an acute and potentially life-threatening complication of CS, as a result of rapid release of vasoactive substances stocked in carcinoid tumour cells, manifesting as severe flushing, bronchospasm, profound hypotension because of haemodynamic instability and arrhythmias [97]. However, there is no clear consensus about the precise definition of carcinoid crisis. This complication more often occurs during stressful procedures, such as anesthesia and/or tumour manipulation during surgery, mimicking an anaphylactoid reaction, and posing a clinical challenge for surgeons and anesthetists [98]. In the perioperative setting, retrospective series have reported incidence of carcinoid crisis in up to 30% of patients CS.[99–100]. Anecdotal reports have also described carcinoid crisis occurrence after repeated abdominal examination of patients with uncontrolled CS [101–102]. Additionally, a case of carcinoid crisis has been previously reported after injection of 6-18F-fluorodihydroxyphenylalanine used as a tracer for positron emission tomography (PET) [103]. From clinical experience, in patients whose carcinoid symptoms are not completely controlled, carcinoid crisis has also been observed following liver biopsy and peptide radionuclide therapy.

Somatostatin analogues have been previously proposed as the standard preventive strategy for carcinoid crisis [104]. Tryptophan replacement was previously suggested as an adjunct therapy [105]. Although the current evidence is limited to small series, the use of infusional octreotide has been proposed to prevent intraoperative carcinoid crisis [69]. However, the literature is controversial in this regard and recent evidence points towards lack of efficacy of somatostatin analogues in preventing carcinoid crisis. A retrospective study indicated that octreotide LAR and preoperative bolus octreotide were not associated with a reduced incidence of perioperative carcinoid crisis [100]. A recently published meta-analysis of retrospective analyses endorsed these findings [106]. A prospective study evaluated the preventive use of a perioperative bolus of 500 μg of octreotide followed by an intraoperative infusion at a rate of 500 μg/hour. Crises occurred at a similar frequency than the historical control of a previous series (30% versus 24%) [99]. Aprotinin, an antifibrinolytic molecule, was not linked to prevention of perioperative carcinoid crisis in patients with CHD [107]. In cases of severe haemodynamic instability, the use of vasopressors and inotropes in conjunction with octreotide appears safe [67]. Overall, we recommend that patients have maximum symptom control before undergoing more aggressive interventions. For those whose symptoms cannot be properly relieved with medical therapy, close monitoring of carcinoid crisis is crucial to avoid life-threatening events.

## Niacin deficiency

The essential amino acid tryptophan is the precursor of both niacin (vitamin B3) and serotonin. The uncontrolled production of serotonin through the two-step enzymatic cascade dependent on tryptophan 5-hydroxylases 1 and 2 and aromatic L-amino acid decarboxylase in carcinoid cells may divert this amino acid stocks from the niacin generation pathway leading to its depletion [108–109]. For this reason, patients with uncontrolled CS are prone to develop niacin deficiency [110]. Pellagra is the clinical syndrome of vitamin B3 deficiency and leads to hypoalbuminaemia, rough scaly skin, angular stomatitis, glossitis, diarrhoea, and encephalopathy. Dermatitis exacerbation may follow exposure to sunlight [111]. Bell and colleagues described a series of 21 patients with CS and detected five subjects with clinical signs of pellagra and two with scleroderma. Both clinical manifestations appeared to occur in patients with more advanced disease and uncontrolled CS [90]. Bouma *et al* retrospectively identified 42 patients with CS with tryptophan deficiency and/or clinical signs of pellagra at the start of niacin supplementation as part of standard care, and he assessed tryptophan levels and niacin status based on the urinary niacin metabolite N^1^-methylnicotinamide (N^1^-MN). They found that pre-supplementation urinary N1-MN levels were lower in 14 of 34 patients as compared with healthy controls and that niacin supplementation normalised this alteration in 12 of them. The majority of patients (86%) received oral nicotinamide at physician’s choice to increase niacin levels [112]. The diagnosis of niacin deficiency in patients with CS is quite rare but important because this is a potentially reversible complication of CS.

## Cognitive impairment and psychiatric disorders

An increasing body of evidence links CS with cognitive disorders. The mechanism likely reflects serotonin depletion in the brain. Chambers and colleagues detected an impairment of cognitive function, especially in verbal memory delayed recall and visual-perceptual function, after analysing 21 patients with CS [113]. In a recent cross-sectional study, 36 patients with small bowel NET were compared with 20 cancer patients with non-neuroendocrine liver metastasis in terms of cognitive function. Patients with CS had worse scores in all cognitive domains, such as initiation, processing speed, visual memory, cognitive efficiency, and delayed verbal recall compared with age/sex/educational-matched controls. They also had significantly delayed recall and marginal slower speed mental flexibility [114]. In another study 15 of 20 patients with CS experienced symptoms of aggressive impulse which were associated with decreased tryptophan levels when compared to controls [115].

On the other hand, treatments aiming at reducing serotonin levels to control CS may be linked to psychiatric disorders. An old inhibitor of the serotonin synthesis, parachlorophenylalanine, although effective in controlling CS was abandoned because of high incidence of psychiatric disorders [116]. In the TELESTAR trial, depressive symptoms occurred more commonly in the higher dose group (500 mg t.i.d.) of telotristat versus the lower dose (250 mg t.i.d) and placebo, i.e. in 6/45 versus 2/45 versus 3/45 patients respectively. All events were mild-to-moderate and resolved while resuming therapy [57, 117]. However, statistical inferences in this regard were not possible because of the low number of events.

It is important to be attentive to psychiatric disorders in patients with CS, particularly in those with uncontrolled carcinoid symptoms. Although the evidence is still insufficient in this regard, we recommend that physicians should actively enquire patients with CS about depressive symptoms as well as sleep disturbances and cognitive dysfunction.

## Muscle wasting and bone density

A potential complication of CS is muscle wasting and proximal myopathy. The largest series of CS do not report symptoms of muscle wasting. However, few case reports have previously documented this association. Berry and colleagues reported a case of CS with severe proximal myopathy in which treatment with cyproheptadine produced symptom improvement in muscular power [118]. A recent cross-sectional study compared 25 patients with CS with 25 healthy controls and could not detect significant differences between cases and controls regarding bone density, bone structure, or bone formation markers (amino-terminal propeptide of type I procollagen and C-terminal telopeptide of type I collagen) [119]. Such muscle wasting is likely resultant from malnourishment associated with uncontrolled CS-induced diarrhoea.

## Diarrhoea

Diarrhoea is an important feature of CS and occurs in up to 75% of patients [6]. Along with flushing and bronchospasm, diarrhoea has constituted a pillar of CS diagnosis [4]. Chronic diarrhoea in CS is typically secretory: largely aqueous and sometimes explosive, possibly leading to mild-to-severe complications, such as dehydration and electrolyte imbalance. [120]. It can be debilitating and incapacitating, with poor QoL being reported by patients with CS and increased frequency of bowel movements [121].

The main causative agent of diarrhoea in CS is probably serotonin, which induces increased secretion through binding to 5-HT_4_ and 5-HT_2A_ receptors, in ileal and sigmoid mucosa respectively [122]. In an experimental model of CS, diarrhoea outbreaks were linked to increased levels of 5-HIAA [123]. Furthermore, serotonin-induced increased gut motility may contribute to diarrhoea in CS patients [124]. Curiously, abrupt cessation of diarrhoea and development of constipation in the context of CS can represent the development of mesenteric fibrosis complication [125].

Antidiarrhoeics (e.g. loperamide) can control mild episodes of diarrhoea. As reviewed by Modlin and colleagues [4], somatostatin analogues, such as octreotide and lanreotide, control diarrhoea in a great proportion of patients (70–80%). Telotristat etiprate, a serotonin synthesis inhibitor, is effective in CS patients with refractory diarrhoea. [57]. A recent case report demonstrated a possible potential of the epigenetic treatment in CS. A patient with chronic refractory diarrhoea because of pulmonary carcinoid had complete resolution of diarrhoea after treatment with RRx-001, an inhibitor of DNA methylation and deacetylation 126].

## Conclusions

**Figure 2.**
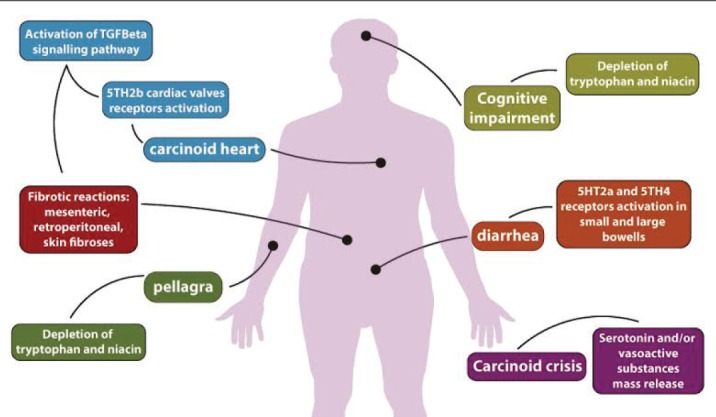
Main complications from CS and proposed pathogenesis.

CS is a classical clinical feature of well-differentiated neuroendocrine tumours, particularly seen in those from midgut sites. This syndrome can lead to clinically important and life-threatening complications provoked by tissue fibrotic degeneration, as well as carcinoid heart disease, mesenteric and retroperitoneal fibroses, and debilitating diarrhoea (Figure 2). Pathogenesis of these complications is likely secondary to serotonin overproduction and the associated activation of signalling pathways such as TGF-ß. It is still controversial whether the control of CS prevents or delays the progression of CHD. However, given the benefit of controlling the CS in terms of patients’ QoL, we recommend that maximum control of carcinoid symptoms be aimed. Medical treatment of established fibrosis is still a clinical challenge as these complications are generally irreversible. Physicians should screen all patients with CS or with asymptomatic 5-HIAA elevation for CHD through an echocardiographic study. It is also important to look for signs of niacin deficiency, a potentially reversible complication of CS, and cognitive and psychiatric disorders. Collaborative, multicentre prospective studies are key to estimate the incidence, prevalence, and clinical course of CHD, to evaluate whether more modern therapies such as everolimus and telotristat impact on CHD and extracardiac fibrosis progression. These studies would also be useful to conduct clinical trials which in turn will help define the optimal management of CS complications associated with neuroendocrine neoplasms.

## Abbreviations

NET: neuroendocrine tumours
CS: carcinoid syndrome
CHD: carcinoid heart disease
SEER: Surveillance, Epidemiology, and End Results
MRI: Cardiac magnetic resonance imaging
5-HIAA: 5-hydroxyindolacetic acid
TGF-β: Transforming growth factor beta
NT-pro-BNP: N-terminal pro-brain natriuretic peptide
